# Monthly Record of Deaths in the City of Buffalo

**Published:** 1854-03

**Authors:** James M. Newman

**Affiliations:** Health Physician


ART. IV.—*Monthly Record of Deaths in the City of Buffalo.* By JAMES M. NEWMAN, M. D., Health Physician.

MESSRS. EDITORS,—I propose, if you deem the subject of sufficient importance, to devote the space necessary to furnish your pages with a monthly abstract of the number and causes of deaths occurring in this city, drawn from the returns made to the Board of Health, and the records kept by the clerk of the Board.

The utility of such reports, from numerous considerations, are, I believe; pretty generally admitted. Their value, however, depends upon their accuracy, and deteriorates rapidly if doubts are permitted to arise of their correctness. The monthly reports heretofore published in this city, have been far from being reliable, and much complaint has, from time to time, been made by the authorities having the matter in charge, of their inability to obtain correct returns, or even an approximation to correctness, from the remissness of the sextons in reporting the number of their burials, and to not a little unpardonable negligence upon the part of physicians in furnishing certificates of the cause of death.

A remedy for these sources of inaccuracy was found by the last Board of Health, in the following ordinance, which was, upon their recommendation, adopted by the Common Council, on the 28th of Nov., 1853. The same is known and designated as sections 10 and 11, of chapter 4, of the ordinances:

" Chap. IV, Sec. 10. No person shall inter within the city of Buffalo, or remove from said city for the purpose of interment, the corpse of any person dying within said city, without first having obtained a permit for that purpose from the city clerk, under the penalty of twenty-five dollars for each and every offense.

"§ 11. The city clerk may grant permission to any person applying therefor to bury the corpse of any person dying within the city, on receiving the certificate of a physician, provided for by the ninth section of this title, or in cases where there was no attending physician, evidence of the cause of such death."

It was not only adopted, but its requirements even immediately, and rigorously put in force, during the balance of their term of office. The effects of its provisions were immediately manifested in the largely increased returns made to the monthly records of mortality in the city. Without any apparent cause of increase, or notable source of mortality in our midst, and, indeed, in a winter remarkable for its geueral health, the number of deaths reported for the month of December, was *one hundred and twenty-nine*, against *sixty-four* for November.

The *ninth section*, referred to above, may, with propriety, be given here, as comprising with the foregoing the whole of the municipal regulations upon the subject of interments within the city, with which physicians are particularly interested, and reads as follows:

"§ 9. The city sexton and every person who may act as sexton in the city of Buffalo, shall obtain before interment, from the attending physician, a certificate of the cause of death, to every person interred by such sexton; and the said physician shall furnish such certificate to such sexton forthwith, after application shall be made to him therefor as aforesaid; and in case of refusal or neglect to comply with the requirements of this section, such sexton and such physician shall forfeit the penalty five dollars for each and every offense. The said certificates shall be filed with the clerk of the Board of Health by the sexton or other person so receiving them, as aforesaid, within one week after their receipt, or such shorter time as the said board may prescribe under a like penalty as aforesaid."

It is, perhaps, unnecessary to add, it is the determination of the present Board of Health to exact as rigorous a compliance with the demands of the above ordinances, as that instituted by our predecessors, and, if necessary, to enforce obedience to its requirements, by the infliction of the penalties annexed.

Errors, it is believed, will now be guarded against, and every confidence may be felt in the perfect reliability of the returns in future.

The record for the month of January, gives the first returns of the city under its new charter, and from within its enlarged boundaries; and forms, consequently, an appropriate period from which to date an effort at a systematic record.

This large increase of territory should be borne in mind by those who may compare the present returns with the last.

In connection with these reports there is a subject to which I would most respectfully beg leave to call the attention of physicians, and that is, to the exercise of greater care upon their part in the return of diseases, and to an effort to introduce a more correct and scientific nomenclature in their certificates. The value of returns so prepared, for future comparison and study, would be much enhanced by very little trouble on their part.

I have before me a copy made from the returns for December. There is not only great confusion in the terms used, and every variety of name employed, but a great proportion given, are totally inadequate to convey any idea of the nosology, or pathology of the disease, which caused death. In many instances the most common, vulgar name is given, which is, perhaps, sufficient to satisfy the public, but which conveys to the physician a very indistinct idea, and often none at all, of the particular cause of death.

Perfect accuracy in this matter, however much to be desired, is, of course, not obtainable, and not to be expected. Many of the returns are made by totally incompetent persons. Some of the dead have had no regular medical attendant; others have been treated by *very irregular* practitioners, and in a mixed population like ours, with many not speaking our language, the subject is environed with additional difficulties. But an approximation to correctness, at least, could be made with a little effort, and would seem worthy the attempt.

REGISTER OF MORTALITY, OF THE CITY OF BUFFALO, FOR THE MONTH OF JANUARY, 1854.

| DISEASES. | No. | Males. | Females. |
|---|---|---|---|
| Abscess, Hepatic, | 1 | 1 | |
| " Psoas, | 1 | 1 | |
| Ascites, | 1 | 1 | |
| Biliary obstruction, | 1 | 1 | |
| Burnt, | 1 | 1 | |
| Cancer of the Breast, | 1 | | 1 |
| Cholera Infantum, | 1 | | 1 |
| Congestion of Brain, | 1 | 1 | |
| " " Lungs, | 1 | | |
| Consumption, | 21 | 6 | 15 |
| Convulsions, | 14 | 9 | 4 |

REGISTER OF MORTALITY—(Continued.)

| DISEASES. | No. | Males. | Females. |
|---|---|---|---|
| Croup, | 5 | 2 | 3 |
| Delirium Tremens, | 1 | 1 | |
| Diarrhœa, | 1 | 1 | |
| Disease of Heart, | 1 | 1 | |
| Enteritis, | 2 | | 2 |
| Fracture of Skull, | 2 | 2 | |
| Hæmoptysis, | 1 | 1 | |
| Hydrocephalus, | 4 | 1 | 3 |
| Hydrops Pectoralis, | 1 | 1 | |
| Inflammation of Brain, | 1 | 1 | |
| " " Throat, | 1 | | 1 |
| Jaundice, | 2 | 1 | 1 |
| Measles, | 4 | 1 | 3 |
| Old Age, | 2 | 1 | 1 |
| Pleurisy, | 1 | 1 | |
| Pneumonia, | 13 | 9 | 4 |
| " Typhoid, | 2 | 2 | |
| Puerperal Inflammation, | 1 | | 1 |
| Scarlet Fever, | 18 | 9 | 8 |
| Scrofula, | 1 | 1 | |
| Small-pox, | 1 | 1 | |
| Still-born, | 8 | | |
| Teething, | 1 | | 1 |
| Typhoid Fever, | 6 | 3 | 3 |
| Uterine Hemorrhage, | 1 | | 1 |
| Unknown, | 20 | 12 | 8 |
| | 145 | | |

| | |
|---|---|
| Males, | 73 |
| Females, | 61 |
| Sex not given, | 11 |
| Total, | 146 |

AGES.

| | | | |
|---|---|---|---|
| Still-born, | 8 | Between 3 years and 6 years, | 11 |
| 1 day, | 3 | " 6 " " 12 " | 2 |
| Between 1 day and 15 days, | 11 | " 12 " " 20 " | 9 |
| " 15 days and 30 days, | 3 | " 20 " " 30 " | 16 |
| " 1 month and 3 months, | 7 | " 30 " " 40 " | 10 |
| " 3 months and 6 months, | 6 | " 40 " " 50 " | 5 |
| " 6 " " 9 " | 6 | " 50 " " 60 " | 5 |
| " 9 " " 12 " | 5 | " 60 " " 70 " | 5 |
| " 1 year and 3 years, | 29 | Ages not given, | 4 |
| Total, | | | 145 |

NATIVITY.

| | | | |
|---|---|---|---|
| American, | 60 | French, | 4 |
| English, | 3 | Canada East, | 1 |
| Scotch, | 3 | Norwegian, | 1 |
| Irish, | 8 | Colored, | 1 |
| German, | 28 | Country not given, | 36 |
| Total, | | | 145 |

Several of the certificates are imperfect in not containing a proper record of the age, sex, and nation of the deceased. A part of the diseases put down as "unknown," are so stated in consequence of the unreliability of the reporters, the certificates being given by unprofessional and unqualified persons.

